# A Ratiometric Fluorescent Sensor Based on Chelation-Enhanced Fluorescence of Carbon Dots for Zinc Ion Detection

**DOI:** 10.3390/molecules28237818

**Published:** 2023-11-28

**Authors:** Guangrong Lu, Zhenzhen Jia, Mengdi Yu, Mingzhen Zhang, Changlong Xu

**Affiliations:** 1 Department of Gastroenterology, The Second Affiliated Hospital, Yuying Children’s Hospital of Wenzhou Medical University, Wenzhou 325000, China; 210257@wzhealth.com; 2 School of Basic Medical Sciences, Health Science Center, Xi’an Jiaotong University, Xi’an 710061, China; jzz324716@stu.xjtu.edu.cn (Z.J.); 18303620993@163.com (M.Y.)

**Keywords:** carbon dots, double-emitting, ratiometric fluorescent sensor, chelation, surface groups

## Abstract

Zinc ion, one of the most important transition metal ions in living organisms, plays a crucial role in the homeostasis of the organism. The disorder of zinc is associated with many major diseases. It is highly desirable to develop selective and sensitive methods for the real-time detection of zinc ions. In this work, double-emitting fluorescent carbon dots (CDs) are prepared by a solvothermal method using glutathione, L-aspartic acid, and formamide as the raw materials. The carbon dots specifically recognize zine ions and produce a decrease in fluorescence intensity at 684 nm and an increase at 649 nm, leading to a ratiometric fluorescent sensor for zinc detection. Through surface modification and spectral analysis, the surface groups including carboxyl, carbonyl, hydroxyl, and amino groups, and C=N in heterocycles of CDs are revealed to synergistically coordinate Zn^2+^, inducing the structural changes in the emission site. The CDs can afford a low limit of detection of ~5 nM for Zn^2+^ detection with good linearity in the range of 0.02–5 μM, showing good selectivity as well. The results from real samples including fetal bovine serum, milk powder, and zinc gluconate oral solution indicated the good applicability of the CDs in the determination of Zn^2+^.

## 1. Introduction

Zinc ion, as one of the most essential transition metal ions, plays a very important role in many biological processes such as neural signaling, gene transcription and expression, cell proliferation, and apoptosis [1,2,3,4]. In living organisms, zinc is mostly bound to proteins, while a small amount of zinc is present in the divalent free state [5,6,7]. An imbalance of zinc ion concentration is closely related to serious diseases, such as amyotrophic lateral sclerosis, ischemic stroke, superficial skin diseases and diabetes [8,9,10]. Moreover, excess zinc in the environment results in a decrease in soil fertility due to the decrease in microbial activity induced by zinc.

To date, a variety of methods have been developed for the detection of zinc ions. The traditional methods include atomic absorption spectroscopy, X-ray fluorescence, and inductively coupled plasma mass spectrometry, all of which are limited in use due to high cost and complicated operation, etc. [11,12,13,14,15]. The probe based on the fluorescent signal for zinc ion detection has been an active topic due to its rapid response, simple operation, and high sensitivity [16,17]. Fluorescence probes for analyzing Zn^2+^ have been constructed to date based on different fluorophores, such as Schiff base [18], naphthalene [19], cyanine [20], fluorescein [21], coumarin [22], pyrene [23], and quinoline [24]. Liu et al. synthesized a series of naphthalene-2-pyridyl hydrazone compounds, which chelate with zinc ions, leading to the enhancement of fluorescence emission. The limit of detection (LOD) for zinc ions reached 0.17 μM [19]. Arghyadeep Bhattacharyya et al. [25] reported that the DIDOP probe, a coumarin-based fluorescent probe for zinc ion detection, can redshift the fluorescence emission peak from 506 nm to 535 nm through charge transfer in the presence of Zn^2+^. However, most fluorescent probes were limited by poor water solubility, low sensitivity, and insufficient selectivity. Hence, it is of significance to construct a rapid, better water solubility, high selectivity, and sensitive probe for the detection of Zn^2+^.

It is worth noting that carbon dots, as a new type of carbon nanomaterial, have the advantages of simple synthesis, low cost, stable fluorescence, high biosafety, and easy solubility in water, etc. [26,27,28,29,30], which has been widely used in analytical detection [31,32,33].

**Scheme 1:**
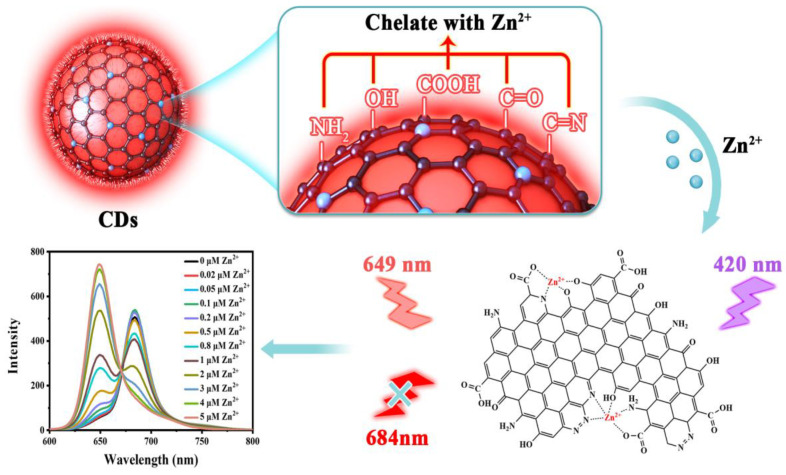
Mechanism illustration of the CDs for the detection of zinc ion.

Herein, a novel kind of red emissive CDs was synthesized through the solvothermal method using glutathione, L-aspartic acid, and formamide as sources. The synthesized CDs are water soluble, well dispersed, and exhibited a red emission of 684 nm under excitation of 420 nm. When Zn^2+^ was added, the fluorescence of the CDs at 684 nm gradually decreased, while the fluorescence intensity at 649 nm gradually increased. Therefore, the ratiometric fluorescent probe with the ratio of fluorescence intensity at 649 nm to that of 689 nm was constructed to evaluate the Zn^2+^ concentration (Scheme 1). Finally, the functional groups (amino, carboxyl, hydroxyl, and carbonyl) on the surface of CDs were passivated separately using chemical modification methods to study the mechanism of chelation between CDs and Zn^2+^.

## 2. Results and Discussion

### 2.1. Characterization of CDs

**Figure 1:**
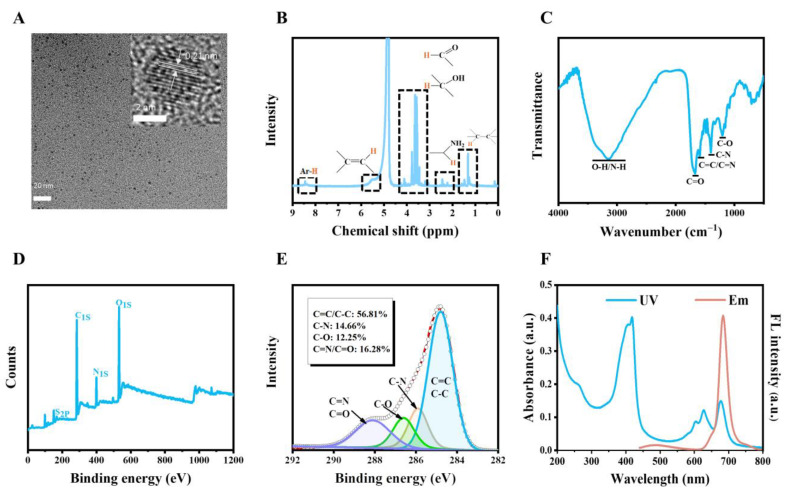
Characterization of CDs. (**A**) The TEM image of CDs. Scale bar: 20 nm. The inset is the HR-TEM image and lattice of the CDs. Scale bar: 2 nm. (**B**) ^1^H NMR spectrum of the CDs. (**C**) FT-IR spectrum of the CDs. (**D**) The XPS results of the CDs. (**E**) High resolution of C 1s with identification of peaks by curve fitting of the CDs. (**F**) The UV spectrum and the fluorescence emission spectrum of CDs (10 μg/mL, ultrapure water).

The carbon dots (CDs) were prepared by a hydrothermal method using glutathione, L-aspartic acid, and formamide as the raw materials. The morphology and structure of CDs were characterized by transmission electron microscopy (TEM), X-ray photoelectron spectroscopy (XPS), nuclear magnetic resonance spectroscopy (NMR), Fourier infrared spectrometer (FT-IR), and Raman spectroscopy. As shown in the transmission electron microscopy image (Figure 1A), the CDs exhibited good dispersibility with a size of 2.27 ± 0.41 nm (Figure S1). The crystalline spacing of CDs was 0.21 nm corresponding to the (100) crystalline surface of graphitic carbon. In the ^1^H-NMR spectrum of CDs (Figure 1B), the peaks in the range of 1.0–1.8 ppm were attributed to ^1^H of aliphatic hydrocarbons, and the peaks in the range of 1.8–2.5 ppm could be ascribed to α-H of amino or carboxyl groups. The peaks in the range of 3.5–3.8 ppm could be attributed to α-H of carbonyl and hydroxyl groups, while the peaks at 5.5 ppm and 7.0–8.5 ppm indicated the presence of alkene and aromatic rings, respectively. The FT-IR spectrum of CDs is shown in Figure 1C. The wide absorption band at 3000–3600 cm^−1^ was attributed to the O-H/N-H stretching vibration, and the peak at 1670 cm^−1^ indicated C=O stretching vibration. The peaks at 1591 cm^−1^, 1402 cm^−1^, and 1200 cm^−1^ were attributed to C=C/C=N, C-N, and C-O, respectively. In the XPS survey of CDs, the peaks at 167.8, 284.9, 400.0, and 532.1 represented the S 2p, C 1s, N 1s, and O 1s, respectively, indicating that CDs were composed of C, N, O, and S with molar ratios of 64.23%, 12.24%, 22.24%, and 1.28%, respectively (Figure 1D). The high-resolution (HR) C 1s was fitted with four peaks (Figure 1E) at 284.8, 285.9, 286.6, and 288.1 eV according to C=C/C-C, C-N, C=O, and O-C=N/C=O, respectively. The HR N 1s was well fitted with three peaks at 398.9, 400.07, and 400.93 eV corresponding to amino, C=N, and N=N, respectively (Figure S2). The above results illustrated that CDs were covered with carboxyl, hydroxyl, amino, and carbonyl groups. The Raman spectrum of CDs exhibited two peaks at 1350 cm^−1^ and 1590 cm^−1^ attributing to the D and G bands, respectively, where the D band represented the disordered defect structure and the G band represented the graphitic structure (Figure S3). The ratio of I(D)/I(G) was 0.958 (Figure S3), suggesting a large portion of defects existed in CDs.

The CDs exhibited a narrow emission peak at 684 nm with a peak width at half height of 28 nm and a shoulder peak at 649 nm, suggesting double emission sites on CDs (Figure 1F). As shown in Figure 1F, The CDs had multiple absorption bands in the UV–Vis range. The absorption band in the range of 200–300 nm can be ascribed to π → π* transition of the aromatic ring of CDs, while the absorption bands at 350–450 nm and 550–750 nm can be assigned to π → π* and n → π* transition of the aromatic π system containing C=N, C=O, and C=S, respectively [27]. In addition, the zeta potential of CDs was −29.23 ± 1.01 mV, indicating the stability of CDs under physiological conditions (Figure S4).

### 2.2. The Properties of CDs

**Figure 2:**
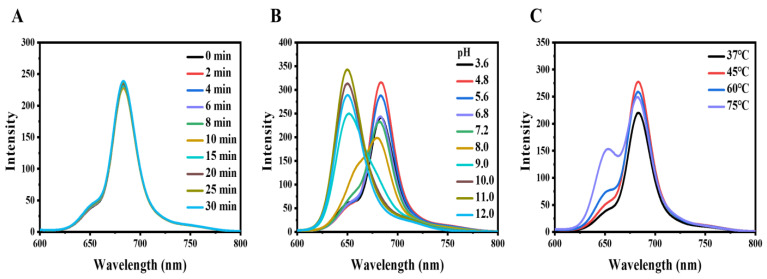
Fluorescence characters of CDs. (**A**) Fluorescence spectra of the CDs under excitation at 420 nm after different irradiation times. (**B**) Fluorescence spectra of the CDs under excitation at 420 nm under different pH values. (**C**) Fluorescence spectra of the CDs under excitation at 420 nm under different temperatures.

Next, the fluorescence responses of CDs toward light, pH, and temperature were investigated. The fluorescence of CDs was relatively stable within 30 min under the continuous excitation of an LED lamp with 800 W (Figure 2A), indicating the good photostability of CDs. The fluorescence spectrum of CDs did not change significantly under the pH ranging from 3.6 to 6.8 except for a slight change in intensity. However, when the pH further increased, the fluorescence spectrum of CDs changed dramatically (Figure 2B). The fluorescence intensity at 649 nm gradually increased with the maximum intensity at pH 11, while that at 684 nm decreased (Figure 2B). The pH-sensitive fluorescence of CDs was assumed to be the protonation/deprotonation and tautomerism between emission site structures [34,35]. Moreover, when the temperature increased from 37 to 75 °C, the fluorescence intensity of CDs at 684 nm decreased while the fluorescence intensity at 649 nm increased (Figure 2C). This phenomenon is more likely to occur at high temperatures and may be attributed to deprotonation, which is consistent with the pH-sensitive fluorescence of CDs.

### 2.3. Colorimetric Fluorescence Detection of Zinc Ion

Firstly, we investigated the effects of a series of ions (Ag^+^, Zn^2+^, Cu^2+^, Ca^2+^, Mg^2+^, Ni^2+^, Fe^2+^, Fe^3+^, Ce^3+^, Au^3+^) on the fluorescence of CDs under the excitation wavelength of 420 nm (Figure S5A). The fluorescence emission at 684 nm of CDs could be quenched by all of these metal ions except Zn^2+^. Interestingly, the fluorescence emission at 649 nm was very sensitive to Zn^2+^. The enhancement factor was 13.3, much more than that of the previous CDs [36]. The fluorescence quantum yield of the CDs was calculated to be 14.09%. Upon addition of Zn^2+^, the CDs-Zn^2+^ complex exhibited reduced fluorescence quantum yield (2.04%).

**Figure 3:**
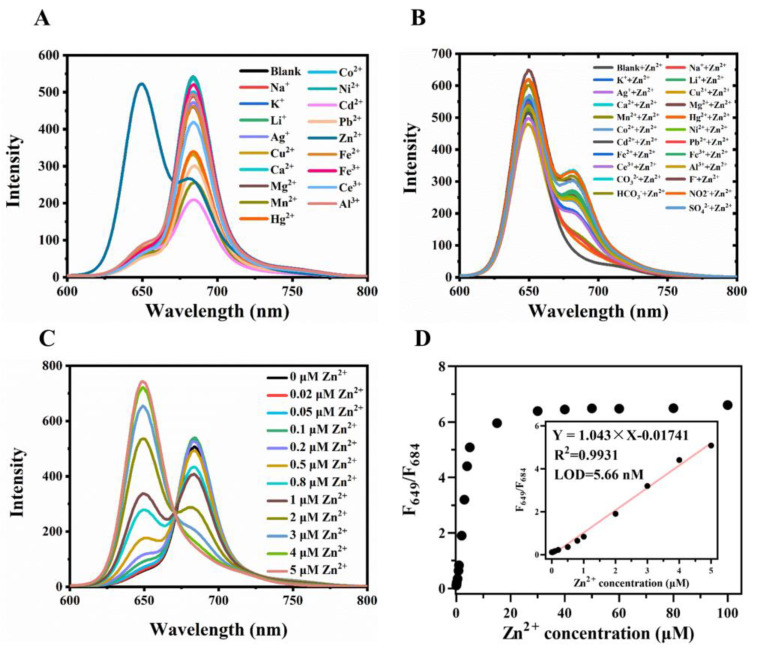
Performance of the CDs for detection of zinc ions. (**A**) Fluorescence spectra of the CDs (10 μg/mL, ultrapure water) upon addition of different metal ions (2 μM) with excitation at 420 nm. (**B**) Fluorescence spectra of the CDs (10 μg/mL, ultrapure water) with excitation at 420 nm after zinc ions (2 μM) and the indicated substrates (2 μM) were added. (**C**) Fluorescence spectra of the CDs (10 μg/mL, ultrapure water) in the presence of zinc ions with different concentrations under excitation at 420 nm. (**D**) The value of the ratio of fluorescence intensity at 649 nm to that at 684 nm (F_649_/F_684_) of the CDs (10 μg/mL, ultrapure water) in the presence of zinc ions with different concentrations (0–100 μM). The inset is the linearity of F_649_/F_684_ to zinc ion concentration.

We also prepared several similar CDs by replacing L-aspartic acid with L-arginine, L(+)-glutamic acid, or L-histidine, producing Arg-CDs, Glu-CDs, and His-CDs, respectively. As shown in Figure S5, all of them displayed two emission peaks at 649 and 684 nm, and the emission at 684 nm could be quenched by metal ions (except Zn^2+^) while the emission at 649 nm was enhanced by Zn^2+^. However, the enhancement factors of Arg-CDs (1.99), Glu-CDs (2.4), and His-CDs (1.95) were much smaller than that of the CDs, indicating that raw materials determine the structure of the emission site of CDs. Considering the high sensitivity of the CDs towards Zn^2+^, the CDs were used to detect Zn^2+^. Firstly, the selectivity and anti-interference of the CDs towards Zn^2+^ were studied. As shown in Figure 3A, among 18 metal ions, only Zn^2+^ could enhance the fluorescence of CDs at 649 nm. Ni^2+^ and Cd^2+^ ions are similar in structure to Zn^2+^ ions; therefore, common fluorescent probes cannot distinguish between them. Here, Ni^2+^ and Cd^2+^ did not affect the fluorescence intensity of CDs at 649 nm (Figure 3A), indicating the excellent selectivity of the CDs. Moreover, the fluorescence intensity at 649 nm of the CDs did not change significantly when zinc ions co-existed with other substances, proving the strong anti-interference ability of CDs (Figure 3B). The stability of CDs-zinc complex was investigated by monitoring the changes in fluorescence intensity against the time at room temperature. The fluorescent intensity of the CDs-zinc complex kept stable at least 8 h at room temperature (Figure S10), indicating its stability and accuracy in Zn^2+^ assay. It is essential to avoid the influence of heavy metals including Cd^2+^, Pb^2+^and Ni^2+^. As the concentration of Zn^2+^ increased from 0.02 to 5 μM, the emission peak of CDs at 649 nm increased while the emission peak at 684 nm gradually decreased (Figure 3C). The ratio of fluorescence intensity at 649 and 684 nm (F_649_/F_684_) was linearly related to zinc ions concentration in the range of 0.02–5 μM, and the limit of detection (LOD) was 5.66 nM (Figure 3D). The conditional binding constant between the CDs and Zn^2+^ was obtained as 5.671 × 10^5^ M^−1^ by the Benesi–Hildebrand equation, which indicates that the CDs possesses better binding ability and fluorescence response to Zn^2+^.

The detection performances of this CDs-based probe, including LOD, linear range, and detection time, were compared with other carbon dot-based probes and small molecule probes. It is evident that the CDs can detect Zn^2+^ rapidly and exhibits high selectivity and sensitivity toward Zn^2+^. Moreover, the CDs also had the advantages of easy synthesis, low cost, good water solubility, and high stability (Table 1).

We also evaluated the binding ability between CDs and zinc ions. Ethylenediaminetetraacetic acid disodium salt dihydrate (EDTA) and citric acid monohydrate (CA) could chelate with Zn^2+^ with high stability constants. We constructed a CDs-Zn^2+^ system, in which the Zn^2+^ (final concentration of 20 μM) was saturated to CDs (final concentration of 10 μg/mL), producing a single emission peak at 649 nm. When the EDTA concentration approached 20 μM, the fluorescence intensity at 649 nm of the CDs-Zn^2+^ system only decreased by ~15% (Figure S6). Under the same condition, the fluorescence of CDs-Zn^2+^ did not change significantly in the presence of CA with a concentration of 20 μM. When the EDTA/CA concentration reached 10 times that of Zn^2+^, i.e., 200 μM, the fluorescence intensity at 649 nm of the CDs-Zn^2+^ system decreased by 37–40% (Figure S6). The above results suggest that the chelating ability of CDs for Zn^2+^ was stronger than that of CA and was comparable to that of EDTA.

### 2.4. Effect of Zinc Ions on the Structure of CDs

Understanding the structure-property relationship and mechanism of sensitive fluorescence of CDs towards metal ions could help rational design different carbon dots to realize desirable sensors for metal ions detection. The UV–Vis absorption spectrum of CDs significantly changed in the presence of Zn^2+^ (Figure S7). The absorption band at 350–450 nm was enhanced while the absorption peak at 677 nm shifted to 640 nm, demonstrating the formation of the ground-state complex (Figure S7). In addition, after combination with Zn^2+^, the zeta potential of the CDs increased from −29.2 mV to −19.0 mV, indicating that the CDs could chelate with multiple Zn^2+^ rather than multiple CDs being cross-linked through Zn^2+^ (Figure S4). Fluorescence lifetime reflects the average time that fluorophore stays excited state after being excited by a photon. It can be seen that with the concentration of zinc ions increasing (0, 0.5, 1, 2 μM), the average fluorescence lifetimes of the CDs at 680 nm were 5.1–5.2 ns. However, the fluorescence lifetimes at 650 nm shortened from 4.8 to 3.5 ns (Table 2 and Figure S8), indicating that Zn^2+^ affected the deexcitation process of the excited state of the emission site (650 nm). Generally, the lone pair electron of amine nitrogen quenches the fluorescence of nearby fluorophores via electron transfer from N to fluorophore*, and zinc ions can coordinate with N, thereby suppressing this quenching and allowing fluorescence to be enhanced [38]. Hence, we speculated that the CDs have two emission sites at 680 nm and 650 nm. The emission of 650 nm was quenched by lone pair electrons of nearby functional groups, such as amine and phenolic hydroxyl groups, while the emission site of 680 nm could transfer to that of 650 nm through deprotonation and coordination with zinc ions.

### 2.5. The Coordination between CDs and Zinc Ions

**Figure 4:**
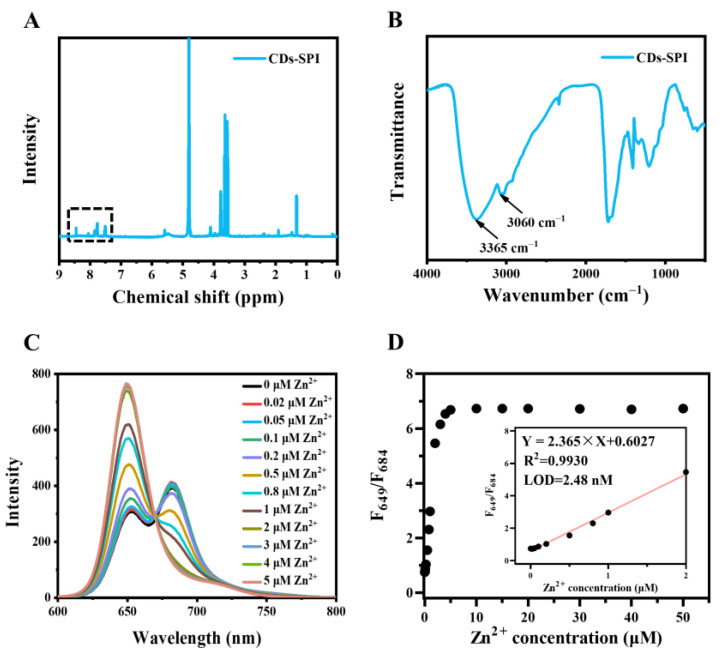
Characterization of CDs-SPI. (**A**) ^1^H NMR spectrum of CDs-SPI. (**B**) FT-IR spectrum of CDs-SPI. (**C**) Fluorescence spectra change in CDs-SPI (10 μg/mL, ultrapure water) upon addition of different concentrations of zinc ions with excitation at 420 nm light. (**D**) F_649_/F_684_ of CDs-SPI (10 μg/mL, ultrapure water) to zinc ions concentration (0–50 μM). The inset is the linearity of F_649_/F_684_ to zinc ions concentration.

To further investigate and reveal the structure-property and fluorescence enhancement mechanism, a series of surface modifications were performed. Firstly, the amino groups were reacted with 4-sulfophenyl isothiocyanate sodium salt (SPI) to study whether the substitution reaction of amino groups affects the coordination between CDs and Zn^2+^. As shown in ^1^H-NMR of CDs-SPI (Figure 4A), the peaks in the range of 7.5–8.5 ppm according to ^1^H in the benzene ring of SPI appeared, indicating that the amino groups of CDs successfully reacted with SPI. As shown in FT-IR of CDs-SPI (Figure 4B), the stretching vibration of unsaturated C-H at 3060 cm^−1^ increased, while the stretching vibration of O-H/N-H shifted from 3147 to 3365 cm^−1^, indicating a decrease in N-H. The fluorescence intensity at 649 nm was significantly enhanced by Zn^2+^ with a saturation concentration of ~5 μM (Figure 4C), much lower than that of CDs (~20 μM), which should be attributed to the steric hindrance of SPI molecular. When CDs-SPI was used to detect zinc ions, the LOD was calculated be 2.48 nM. The F_649_/F_684_ of CDs-SPI toward Zn^2+^ has a good linear relationship (Figure 4D) in the range of 0.02–2 μM. Therefore, it can be speculated that the substitution reaction of amino groups did not affect the coordination effect between CDs and Zn^2+^.

**Figure 5:**
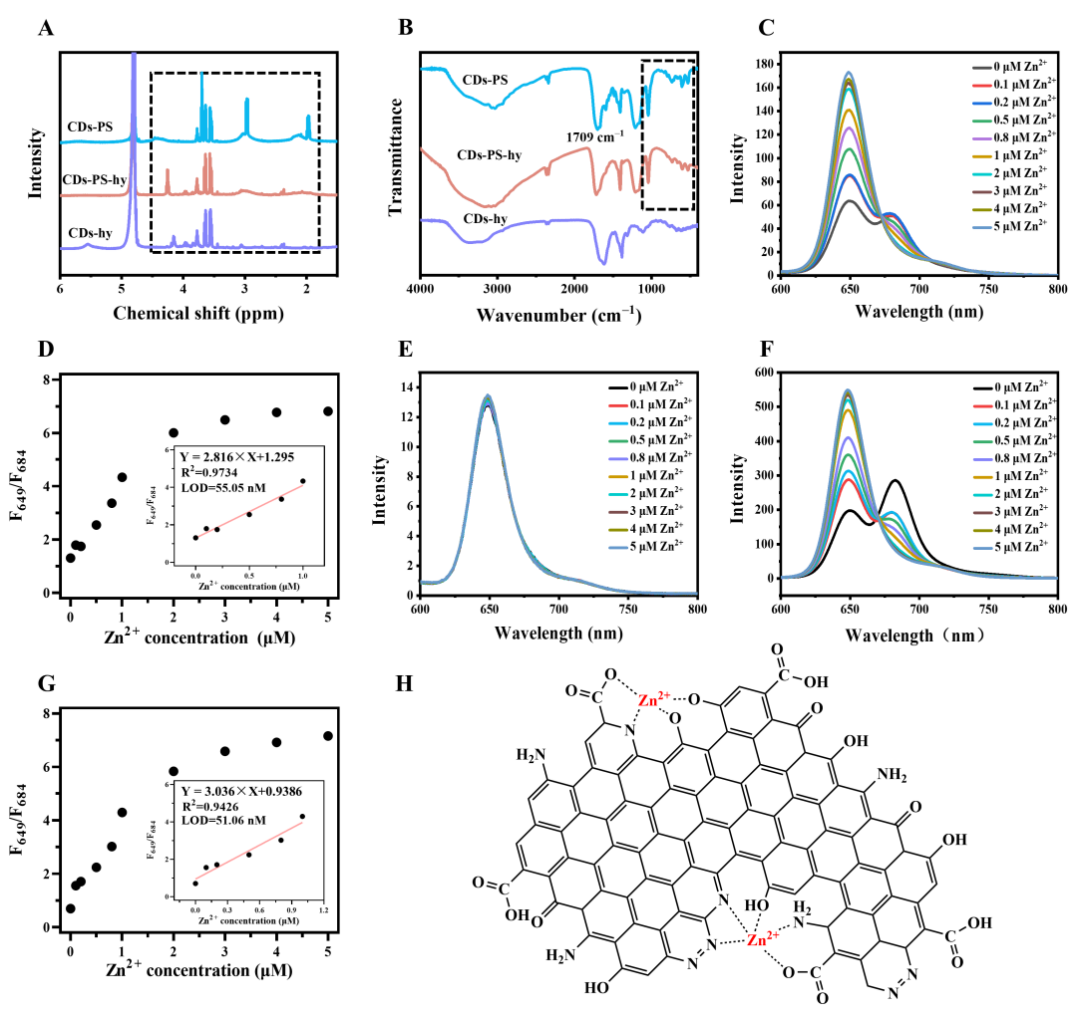
Characterization of CDs-PS, CDs-PS-hy and CDs-hy. (**A**) The ^1^H NMR spectra of CDs-PS, CDs-PS-hy, and CDs-hy. (**B**) The FT-IR spectra of CDs-PS, CDs-PS-hy, and CDs-hy. (**C**) Fluorescence spectra change in CDs-PS (10 μg/mL, ultrapure water) upon addition of different concentrations of zinc ions with excitation at 420 nm light. (**D**) F_649_/F_684_ of CDs-PS (10 μg/mL, ultrapure water) to zinc ions concentration (0–5 μM). The inset is the linearity of F_649_/F_684_ to zinc ions concentration. (**E**) Fluorescence spectra change in CDs-PS-hy (10 μg/mL, ultrapure water) upon addition of different concentrations of zinc ions with excitation at 420 nm light. (**F**) Fluorescence spectra change in CDs-hy (10 μg/mL, ultrapure water) upon addition of different concentrations of zinc ions with excitation at 420 nm light. (**G**) F_649_/F_684_ of CDs-hy (10 μg/mL, ultrapure water) to zinc ions concentration (0–5 μM). The inset is the linearity of F_649_/F_684_ to zinc ions concentration. (**H**) Expected high affinity and selectivity for zinc ions.

We used 1,3-propane sulfonate (PS) to passivate the amino, carboxyl, and hydroxyl groups on the surface of CDs [42]. The amino, carboxyl, and hydroxyl react with PS to produce secondary amine, ester, and ether, respectively, while ester is easily hydrolyzed under alkaline conditions [42,43]. By hydrolyzing the CDs-PS with NaOH, CDs with the hydroxyl and amino groups passivated could be obtained. After being modified by PS, the new peaks at 2.0, 3.0, and 4.4 ppm appeared in the ^1^H-NMR of CDs-PS corresponding to the ^1^H of β, α, γ of -SO_3_^-^ and the decreased in that of CDs-PS-hy (Figure 5A). In the FT-IR spectrum of CDs-PS (Figure 5B), the stretching vibration shifted from 1670 cm^−1^ to 1709 cm^−1^, indicating the formation of an ester. Meanwhile, the characteristic absorption peaks of sulfonic acid groups appeared at 530, 604, and 1046 cm^−1^ in the FT-IR spectra of CDs-PS and decreased in that of CDs-PS-hy (Figure 5B). Of note, the fluorescence intensity at 649 nm of CDs-PS was higher than that at 684 nm (Figure 5C), which might be due to the passivation of functional groups inhibiting the electron transfer between them and the emission site at 649 nm. It could also be seen that the response of CDs-PS toward zinc ions was much less sensitive than CDs. The linear range of CDs-PS towards Zn^2+^ was 0.1–1 μM and the LOD was 55.05 nM (Figure 5D). Taken together, considering the lesser effect of the substitution reaction of amino groups on the coordination between CDs and Zn^2+^, the carboxyl and hydroxyl groups mainly facilitate the coordination between CDs and Zn^2+^ and boost the detection of zinc ions.

After hydrolysis under 0.5 M NaOH solution, the carboxyl group was released. The emission peak at 684 nm of CDs-PS-hy disappeared, leaving only the peak at 649 nm. The fluorescence intensity of CDs-PS-hy was also significantly decreased compared to CDs and CDs-PS, while CDs-PS-hy did not show a response to Zn^2+^ (Figure 5E). In previous work, we found that a 0.5 M NaOH solution had an irreversible effect on the structure of CDs, we treated CDs with the same hydrolytic process as a control, and the resulting sample was named CDs-hy. The structural difference between CDs-hy and CDs-PS-hy was that the hydroxyl and amino groups of CDs-PS-hy were passivated while that of CDs-hy were not. The fluorescence intensity at 684 nm of CDs-hy was slightly higher than that at 649 nm compared to CDs, this confirmed that the NaOH solution would affect the structure of CDs (Figure 5F). Unlike CDs-PS-hy did not show a response to Zn^2+^, CDs-hy exhibited a good linear relationship with Zn^2+^ concentration (0.1–1 μM) and achieved a LOD of 51.06 nM (Figure 5G). Therefore, the hydroxyl of CDs plays a crucial role in chelating with Zn^2+^. Since the carboxyl group could coordinate with Zn^2+^, we tested the fluorescence response of CDs to Zn^2+^ at different pH (Figure S9). When the pH values increased from 3.6 to 8.0, the fluorescence emission spectrum of CDs remained relatively stable. While the fluorescence intensity at 649 nm of CDs increased significantly in the presence of Zn^2+^ (20 μM) and reached a plateau after pH 5.6 (Figure S9A,B). The pK_a_ of the carboxyl group is usually in the range of 4–5 [35], the same range as the fluorescence enhancement of CDs in the presence of Zn^2+^, indicating that carboxyl groups of CDs also chelated with Zn^2+^. We speculated that a variety of surface functional groups of the CDs can chelate with Zn^2+^ (Figure 5H), which would allow enhanced selectivity for Zn^2+^ over other metal ions such as Ni^2+^.

**Figure 6:**
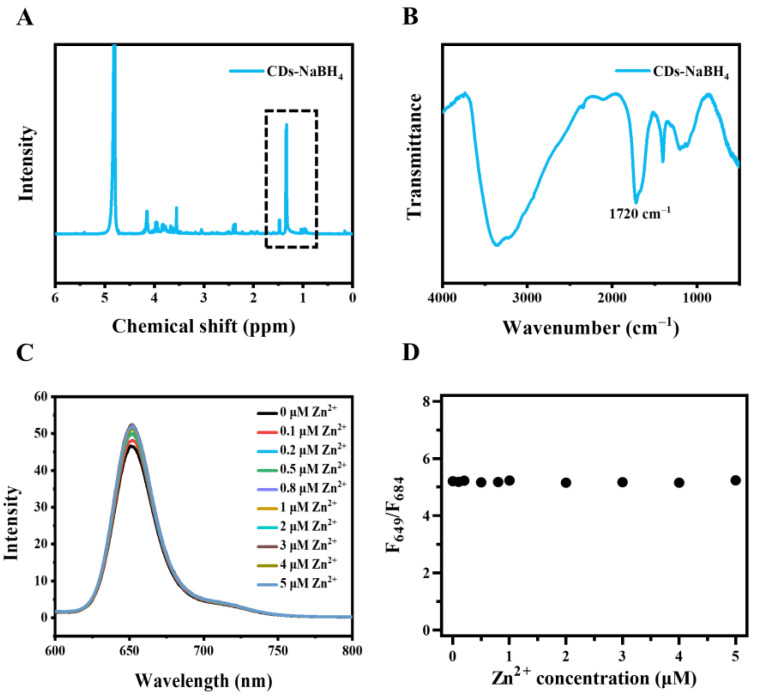
Characterization of CDs-NaBH_4_. (**A**) The ^1^H NMR spectrum of CDs-NaBH_4_. (**B**) The FT-IR spectrum of CDs-NaBH_4_. (**C**) Fluorescence spectra change in CDs-NaBH_4_ (10 μg/mL, ultrapure water) upon addition of different concentrations of zinc ions with excitation at 420 nm light. (**D**) F_649_/F_684_ of CDs-NaBH_4_ (10 μg/mL, ultrapure water) to zinc ions concentration (0–5 μM).

Sodium borohydride is a common reductant that reduces C=O and C=N. As demonstrated in ^1^H-NMR of CDs reduced by NaBH_4_ (CDs-NaBH_4_), a significant increase in the peaks in the range of 0.8–1.5 ppm was observed, indicating that the C=O and C=N on the surface of CDs were reduced to C-O and C-N, respectively (Figure 6A). Meanwhile, the characteristic peaks of C=O at 1720 cm^−1^ and C=N at 1591 cm^−1^ in the FT-IR spectrum of CDs-NaBH_4_ (Figure 6B) decreased significantly, confirming the successful passivation of C=O and C=N. As a result, only the peak at 649 nm remained for CDs-NaBH_4_, and the intensity was significantly lower than that of CDs (Figure 6C), suggesting that C=O and C=N might be the emission site of CDs with a wavelength of 684 nm. There was no linear relationship between F_649_/F_684_ and zinc ion concentration (Figure 6D). It is speculated that the C=O, C=N, hydroxyl, and carboxyl groups on the surface of CDs chelate with Zn^2+^, decreased the electron transfer between these functional groups and the emission site of 649 nm of CDs, and then enhanced the fluorescence.

### 2.6. Detection of Zinc Ions in Complex Real Samples

**Figure 7:**
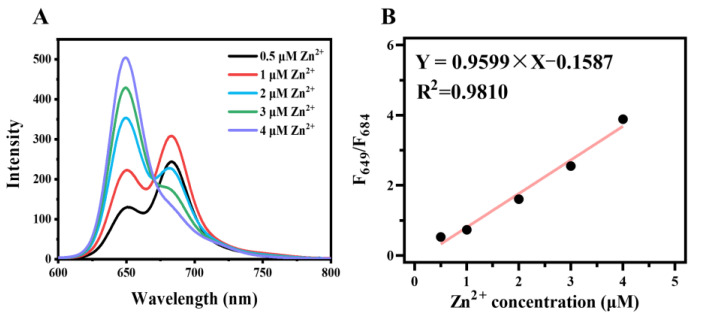
The application of CDs in real samples. (**A**) Fluorescence spectra change in CDs (10 μg/mL, 1.2 mg/mL of milk powder solution) at 420 nm excitation upon addition of different concentrations of zinc ions. (**B**) The linearity of F_649_/F_684_ of CDs (10 μg/mL, 1.2 mg/mL of milk powder solution) to zinc ions concentration.

To evaluate the performance of CDs for Zn^2+^ detection in real samples, the determination of Zn^2+^ in milk powder, zinc gluconate oral solution, and fetal bovine serum was performed. The zinc content in the milk powder measured by CDs was 2.67 ± 0.08 mg/100 g milk powder, which is consist of the labeled content of 2.8 mg/100 g. In addition, a solution with a zinc ions concentration of 0.5 μM was prepared according to the parameters on the package of milk powder, and then Zn(NO_3_)_2_ was added to tune the Zn^2+^ concentrations to 0.5, 1.0, 2.0, 3.0, and 4.0 μM. In milk powder solution, as the concentration of Zn^2+^ increased, the emission peak of CDs at 649 nm also enhanced while the emission peak at 684 nm gradually decreased, which was similar that of to the ultrapure water detection environment (Figure 7A). The relationship between F_649_/F_684_ of CDs in milk powder solution and zinc ions concentration is shown in Figure 7B. The slope of 0.9599 in milk powder solution was close to that of 1.043 in ultrapure water, indicating that CDs can be applied to the detection of zinc ions in complex environments.

Different brands of zinc gluconate oral solution were nitrated, neutralized, and diluted with ultrapure water. Then, the resultant samples were assayed separately with CDs. As shown in Table 3, the quantities of these zinc gluconate oral solutions were determined to be 0.49, 0.47, and 0.51 mg/mL, respectively, with the RSD (*n* = 3) values of 4.1–5.3%, which are consistent with the package calibration (0.5 mg/mL). Fetal bovine serum was also nitrated and underwent rotary evaporation to remove the solvent, then diluted with ultrapure water. For the one fetal bovine serum, the Zn^2+^ concentration were measured by CDs and inductively coupled plasma mass spectrometry (ICP-MS). The results are 1.573 × 10^−3^ mg/mL and 1.561 × 10^−3^ mg/mL, respectively, indicating the accuracy of CDs for Zn^2+^ detection.

## 3. Materials and Methods

### 3.1. Reagents, Materials and Instruments

For details, see the Supplementary Materials.

### 3.2. Synthesis

#### 3.2.1. Synthesis of CDs

First, 1.2 g of glutathione and 1.2 g of L-aspartic acid were dissolved in 40 mL of formamide in a Teflon-lined autoclave. The mixture was heated at 160 °C for 8 h. After cooling to room temperature, the reaction mixture was dialyzed for 7–9 d, and further filtered by 0.22 µm BIOSHARPP membrane filters. By freeze-drying the above solution, the CDs powder was obtained.

#### 3.2.2. Synthesis of CDs-SPI

An amount of 5 mg of CDs and 5 mg of 4-sulfophenyl isothiocyanate sodium salt monohydrate were mixed with 7 mL of carbonation buffer (pH = 9). After reaction for 8 h at 4 °C, the resulting mixture was dialyzed in ultrapure water for 3 d.

#### 3.2.3. Synthesis of CDs-PS

A volume of 1 mL of CDs solution (5 mg/mL) and 1 g of 1,3-propane sultone (PS) were mixed with 10 mL of 1, 4-dioxane. Then, 1 mL triethylamine was added to this mixture. After stirring for 24 h at 40 °C, the mixture underwent rotary evaporation to remove the organic solvent. The resultant mixture was dispersed in ultrapure water and dialyzed in 0.1 M NaCl solution for 1 d to remove triethylamine salt through ion exchange. The product was obtained by dialysis in ultrapure water for 3 d.

#### 3.2.4. Synthesis of CDs-PS-hy

An amount of 5 mg of CDs-PS was dissolved in 10 mL NaOH (0.5 M). After stirring for 24 h at 40 °C, the mixture was neutralized with hydrochloric acid (0.1 M). The produce was obtained by dialysis in ultrapure water for 3 d.

#### 3.2.5. Synthesis of CDs-hy

An amount of 5 mg of CDs was dissolved in 10 mL NaOH solution (0.5 M). After stirring for 24 h at 40 °C. The mixture was neutralized with hydrochloric acid (0.1 M). The resultant mixture was dialyzed in ultrapure water for 3 d.

#### 3.2.6. Synthesis of CDs-NaBH_4_

An amount of 5 mg of CDs was added to 10 mL of NaBH_4_ solution (0.5 M), and the mixture was stirred for 24 h at room temperature. The resultant mixture was neutralized with hydrochloric acid (0.1 M) and then dialyzed in ultrapure water for 3 d.

### 3.3. Effect of Metal Ions on the Fluorescence of CDs

A volume of 100 μL of CDs with a final concentration of 10 μg/mL and 100 μL of different metal ions (Ag^+^, Zn^2+^, Cu^2+^, Ca^2+^, Mg^2+^, Ni^2+^, Fe^2+^, Fe^3+^, Ce^3+^, Au^3+^) with a final concentration of 1 mM were mixed evenly. Then, the fluorescence spectra of mixtures were recorded in the emission wavelength range from 600 nm to 800 nm at the excitation wavelength of 420 nm. The procedure was duplicated three times.

### 3.4. Fluorescence Stability Test of CDs

The CDs solution (10 μg/mL) was handled with different illumination times (0, 2, 4, 6, 8, 10, 15, 20, 25, and 30 min), pH values (3.6, 4.8, 5.6, 6.8, 7.2, 8, 9, 10, 11 and 12) or temperatures (37, 45, 60, and 75 °C). Then, the fluorescence spectra were recorded at the excitation wavelength of 420 nm. The procedure was duplicated three times.

### 3.5. Detection of Zinc Ions by CDs

First, 100 μL of CDs with a final concentration of 10 μg/mL and 100 μL of zinc ions with different concentrations were mixed and vortexed. The fluorescence spectra of mixtures were recorded at the excitation wavelength of 420 nm, respectively. The limit of detection (LOD) was calculated via the 3σ principle.

### 3.6. Detection of Zn^2+^ Levels in Samples

A amount of 1.2 mg milk powder was dissolved thoroughly in 1 mL ultrapure water. Different concentrations of Zn^2+^ (0.5, 1, 2, 3, and 4 μM) were spiked into it. Then, the CDs were added to analyze the Zn^2+^ concentration according to the above steps

Different brands of zinc gluconate oral solution (0.5 mL) were mixed with nitric acid (3 mL) reacted at 140 °C for 4 h, and then neutralized with sodium hydroxide (0.1 M), then volumized to 50 mL. The obtained mixture was diluted 40 times with ultrapure water. After that, 10 μL of CDs solution (1 mg/mL) was added for the detection of Zn^2+^.

Fetal bovine serum (1 mL), nitric acid (1 mL), and hydrogen peroxide solution (0.5 mL) were mixed and reacted at 120 °C for 30 min. The resultant mixture underwent rotary evaporation to remove the solvent, and then was dispersed in 10 mL ultrapure, underwent rotary evaporation to remove the solvent, repeated three times. The mixture was dispersed in 10 mL ultrapure, and diluted 15 times with ultrapure water (1 mL). Finally, 10 μL of CDs solution (1 mg/mL) was added for the detection of Zn^2+^. The procedure was duplicated three times.

## 4. Conclusions

In conclusion, this work strategically synthesized novel red emissive CDs with superior optical properties by a hydrothermal method using glutathione, L-aspartic acid, and formamide as raw materials. The formed CDs displayed a high aqueous solubility with a quantum yield value of 14.09%. Based on the chelation between CDs and Zn^2+^, the CDs had been applied to detect Zn^2+^. The CDs exhibited high selectivity and sensitivity toward Zn^2+^, and could rapidly chelate with Zn^2+^ to achieve a low LOD of 5 nM within a broad linear range of 0.02–5 μM. And the CDs were also used for quantitative analysis of Zn^2+^ in real samples, including milk powder, zinc gluconate oral solution, and fetal bovine serum. Moreover, through surface modifications and spectral analysis, we revealed that the amino, carboxyl, hydroxyl, and C=N/C=O on the surface of CDs chelate with Zn^2+^, and inhibited the electron transfer between functional groups and the emission site of the CDs, which enhanced the fluorescence intensity at 649 nm and decreased at 684 nm due to the coordination. This work brings to light CDs with highly selective sensing for Zn^2+^, identifies the chelation-enhanced fluorescence mechanism of CDs, and realizes the detection of Zn^2+^ in real samples.

## Figures and Tables

**Table 1:** Comparison of different fluorescence probes for the detection of zinc ions.

Probe	Linear Range	Response Time	LOD	Ref.
DPA-P-DTC-QDs	0.9–16 μM	9 min	0.7 μM	[37]
CdSe-ZnS QD	5–500 μM	3 min	2.4 μM	[38]
CDs	250–600 μM	Immediate detection	0.36 mM	[32]
N-S CDs	0.01–1 μM	40 min	5.0 nM	[36]
DCDs	2.5–50 μM	5 min	1.2 μM	[4]
BIPP	0.1–1 μM	Not mentioned	23.6 nM	[39]
Probe	0–10 μM	Less than 20 s	15.3 nM	[9]
ZN-2	0–10 μM	20 min	0.74 μM	[21]
L	0.16–10 μM	Not mentioned	0.15 μM	[40]
HL_1_	Not mentioned	Not mentioned	0.14 μM	[41]
CDs	0.02–5 μM	Immediate detection	5.66 nM	This study
CDs-SPI	Ultrapure water	Immediate detection	2.48 nM	This study

**Table 2:** Fluorescence lifetimes of CDs at 650 nm and 680 nm with increasing zinc ion concentration.

	τ_1_ (ns)	τ_2_ (ns)	τ_3_ (ns)	τ_ave_ (ns)
CDs + 0 μM Zn^2+^-650	1.7899 (4.85%)	4.5394 (90.40%)	13.0837 (4.75%)	4.8119
CDs + 0.5 μM Zn^2+^-650	1.9599 (14.10%)	4.3687 (80.84%)	12.4657 (5.06%)	4.4388
CDs + 1 μM Zn^2+^-650	1.9775 (23.41%)	4.1601 (71.93%)	12.9282 (4.66%)	4.0577
CDs + 2 μM Zn^2+^-650	1.8694 (28.03%)	3.5503 (67.42%)	12.9373 (4.55%)	3.5063
CDs + 0 μM Zn^2+^-680		4.7963 (96.09%)	15.5196 (3.91%)	5.2156
CDs + 0.5 μM Zn^2+^-680		4.7806 (95.75%)	14.0733 (4.25%)	5.1755
CDs + 1 μM Zn^2+^-680		4.7104 (94.37%)	12.2045 (5.63%)	5.1323
CDs + 2 μM Zn^2+^-680	1.5277 (1.77%)	4.7273 (93.66)	14.4138 (4.57%)	5.1133

**Table 3:** Measurement of Zn^2+^ in zinc gluconate oral liquids.

Sample	Original (mg/mL)	Measured (mg/mL)	RSD% (*n* = 3)
1	0.5	0.49	4.1
2	0.5	0.47	4.6
3	0.5	0.51	5.3
4	1.561 × 10^−3^ (ICP-MS)	1.57 × 10^−3^	0.42

Note: 1: 999 zinc gluconate oral solution, 2: Dinggui zinc gluconate oral solution, 3: San jing zinc gluconate oral solution, and 4: Fetal bovine serum from ExCell Bio (Shanghai, China).

## Data Availability

Data are available online or from the author.

## Supplementary Materials

The following supporting information can be downloaded at: https://www.mdpi.com/article/10.3390/molecules28237818/s1, Figure S1. Characterization of CDs. The particle size distribution of CDs. Figure S2. Characterization of CDs. High-resolution of N 1s with identification of peaks by curve fitting of the CDs. Figure S3. Characterization of CDs. Roman spectrum of CDs. Figure S4. Characterization of CDs. Zeta potential of CDs and CDs+Zn^2+^, CDs (10 μg/mL, ultrapure water), Zn^2+^ (20 μM). Figure S5. Fluorescence response of different carbon dots to metal ions. The fluorescence spectra change of (A) GSH-Asp-CDs. (B) GSH-His-CDs. (C) GSH-Arg-CDs. (D) GSH-Glu-CDs (10 μg/mL, ultrapure water) upon addition of different metal ions (Ag^+^, Zn^2+^, Cu^2+^, Ca^2+^, Mg^2+^, Ni^2+^, Fe^2+^, Fe^3+^, Ce^3+^, Au^3+^, all at 1 mM) with excitation at 420 nm light. Figure S6. The binding ability between CDs and zinc ions. (A) Fluorescence spectra of CDs (10 μg/mL, ultrapure water) under 420 nm excitation after adding different concentrations of EDTA and 20 μM Zinc ions. (B) F649/F684 of CDs (10 μg/mL, ultrapure water) and 20 μM Zinc ions versus EDTA concentration (10–1000 μM). (C) Fluorescence spectra of CDs (10 μg/mL, ultrapure water) under 420 nm excitation after adding different concentrations of CA and 20 μM Zinc ions. (D) F649/F684 of CDs (10 μg/mL, ultrapure water) and 20 μM Zinc ions versus CA concentration (10–1000 μM). Figure S7. The absorption spectra of CDs. UV-Vis absorption spectra change of CDs (10 μg/mL, ultrapure water) upon addition of different concentrations of zinc ions. Figure S8. The fluorescence lifetimes of CDs. The fluorescence lifetimes of CDs (10 μg/mL, ultrapure water) at 650 nm and 680 nm for different Zinc ions concentrations. (A) 0 μM Zn^2+^. (B) 0.5 μM Zn^2+^. (C) 1 μM Zn^2+^. (D) 2 μM Zn^2+^. Figure S9. The fluorescence changes of CDs under different pH. (A) Fluorescence spectra of CDs (10 μg/mL) under excitation at 420 nm light with different pH values. (B) Fluorescence spectra of CDs (10 μg/mL) at different pH values with 20 μM Zinc ions under excitation at 420 nm light. (C) F649/F684 of CDs (10 μg/mL) with different pH values (blue), F649/F684 of CDs (10 μg/mL) at different pH values with 20 μM Zinc ions (red). Figure S10. The stability of CDs. The value of the ratio of fluorescence intensity at 649 nm to that at 684 nm (F649/F684) of the CDs (10 μg/mL, ultrapure water) in the presence of zinc ions against time. Figure S11. Benesi–Hildebrand plot of the CDs with Zn^2+^.

## Abbreviations

Zn^2+^, zinc ion; CDs, carbon dots; LOD, limit of detection; EDTA, ethylenediaminetetraacetic acid disodium salt dihydrate; CA, citric acid monohydrate; SPI, 4-sulfophenyl isothiocyanate sodium salt monohydrate; PS, 1,3-propane sultone; TEM, transmission electron microscopy; XPS, X-ray photoelectron spectroscopy; NMR, nuclear magnetic resonance spectroscopy; FT-IR, Fourier infrared spectrometer; UV–Vis, ultraviolet and visible spectrophotometry.
