# Supplementary material for: A Ratiometric Fluorescent Sensor Based on Chelation-Enhanced Fluorescence of Carbon Dots for Zinc Ion Detection
Source: Molecules. 2023 Nov 28;28(23):7818. doi: 10.3390/molecules28237818 (PMC10708225; doi:10.3390/molecules28237818)
Supplement: Supplementary file 1 [file molecules-28-07818-s001.zip › molecules-2664877-supplementary.pdf]

Supplementary Material

Experimental Procedures

Reagents and materials

L-aspartic acid, L-arginine, L(+)-glutamic acid, L-histidine, formamide, glutathione (reduced), 1,3-propane sulfonate (PS), copper sulfate pentahydrate ($CuSO_4 \cdot 5H_2O$), lithium chloride (LiCl), aluminum chloride hexahydrate ($AlCl_3 \cdot 6H_2O$), magnesium chloride ($MgCl_2$) and gold chloride trihydrate ($HAuCl_4 \cdot 3H_2O$) were purchased from Aladdin Chemical Reagent Co., Ltd (Shanghai, China). 1, 4-dioxane, cerium nitrate hexahydrate ($Ce(NO)_3 \cdot 6H_2O$), calcium chloride ($CaCl_2$), potassium chloride (KCl), cobalt chloride hexahydrate ($CoCl_2 \cdot 6H_2O$), Citric acid monohydrate (CA), sodium borohydride ($NaBH_4$), silver nitrate ($AgNO_3$) and iron chloride hexahydrate ($FeCl_3 \cdot 6H_2O$) were purchased from Sinopharm Chemical Reagent Co., Ltd (Shanghai, China). 4-sulfophenyl isothiocyanate sodium salt monohydrate was purchased from Sigma-Aldrich Co., Ltd (Shanghai, China). Sodium chloride (NaCl), sodium hydroxide (NaOH), and sodium hydrogen carbonate ($NaHCO_3$) were purchased from Tianli Chemical Reagent Co., Ltd (Tianjin, China). Zinc nitrate hexahydrate ($Zn(NO_3)_2 \cdot 6H_2O$) was purchased from Tianjin Kemiou Chemical Reagent Co., Ltd (Tianjin, China). Zinc chloride ($ZnCl_2$) and mercuric nitrate monohydrate ($HgN_2O_6 \cdot H_2O$) were purchased from Shanghai Macklin Biochemical Co., Ltd (Shanghai, China). Triethylamine (TEA) was purchased from Energy Chemical Co., Ltd (Shanghai, China). Iron chloride tetrahydrate ($FeCl_2 \cdot 4H_2O$) was purchased from Tianjin Guangfu Technology Development Co., Ltd (Tianjin, China). Chromium nitrate tetrahydrate ($Cd(NO_3)_2 \cdot 4H_2O$) was purchased from Tianjin Guangfu Fine Chemical Research

Institute (Tianjin, China). Manganese chloride tetrahydrate ($MnCl_2 \cdot 4H_2O$) was purchased from Fuchen Chemical Reagent Co., Ltd (Tianjin, China). Lead nitrate ($Pb(NO_3)_2$) was purchased from Tianjin Hongyan Chemical Reagent Factory (Tianjin, China). Nickel chloride hexahydrate ($NiCl_2 \cdot 6H_2O$) was purchased from Damao Chemical Reagent Factory (Tianjin, China). Ethylenediaminetetraacetic acid disodium salt dihydrate (EDTA) was purchased from Bo far Tyrone Biological Technology Co., Ltd (Beijing, China). Fetal bovine serum was purchased from ExCell Bio Co., Ltd (Shanghai, China). Hydrochloric acid (HCl), nitric acid (HNO_3, 65%~68%) , and hydrogen peroxide solution (H_2O_2, 30%) were purchased from local supplies. All aqueous solutions were prepared with deionized water (18.2 MΩ·cm, Millipore).

Instruments

Thermo Fisher Nicolet 5700 and Thermo Fisher DXR2xi were used to measure FTIR spectra and Roman spectra, respectively. X-ray photoelectron spectroscopy was obtained by a Thermo Fisher ESCALAB Xi$^+$. SHIMADZU UV-2700 Spectrophotometer was used to measure UV-Vis absorption spectra. An AVANCE III HD spectrometer (600 MHz) was employed to perform the proton magnetic resonance (^{1}H NMR) spectra. A Malvern Zetasizer Nano ZSE was used to record the Zeta potential. Transmission electron microscopy (TEM) and high-resolution TEM images were obtained from a JEM-2100 microscope. The fluorescence spectra were obtained from a HITACHI-F4700. Fluorescence lifetime was recorded on Edinburgh FLS9 at room temperature. American Agilent ICPOES730 was used to measure zinc ions content in fetal bovine serum.

Supplementary Figures

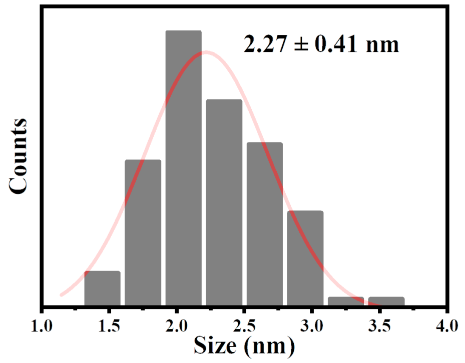

Figure S1. Characterization of CDs. The particle size distribution of CDs.

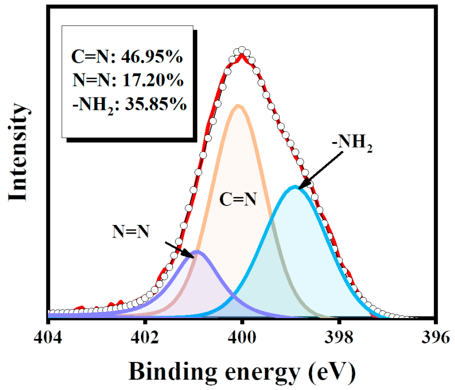

Figure S2. Characterization of CDs. High-resolution of N 1s with identification of peaks by curve fitting of the CDs

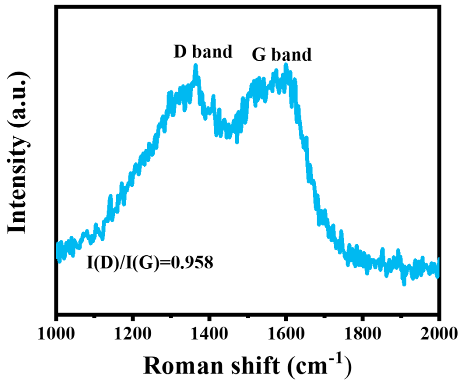

Figure S3. Characterization of CDs. Roman spectrum of CDs.

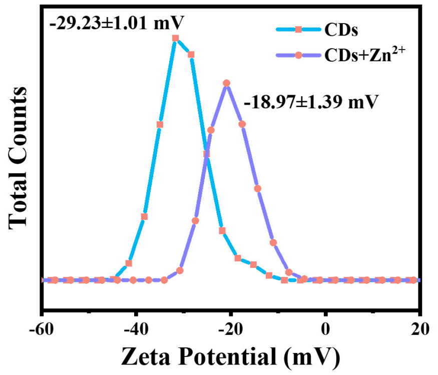

Figure S4. Characterization of CDs. Zeta potential of CDs and CDs+Zn^{2+}, CDs (10 μg/mL, ultrapure water), Zn^{2+} (20 μM).

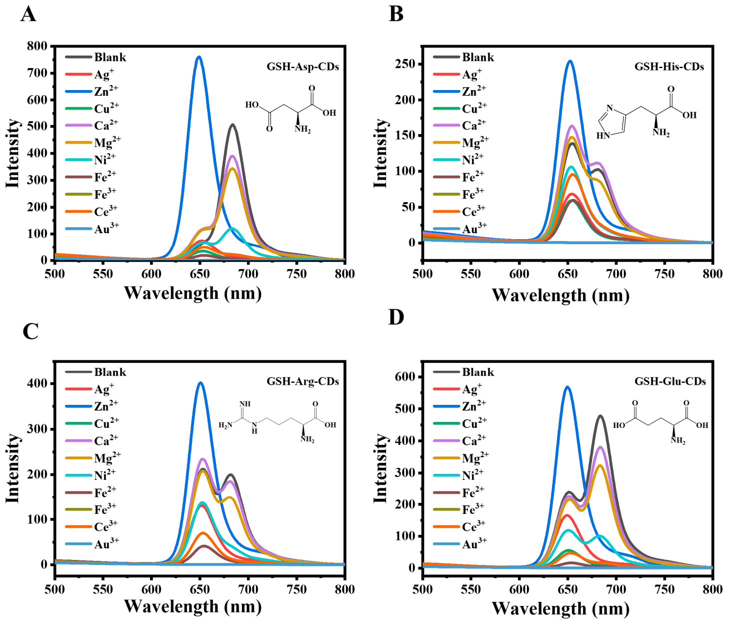

Figure S5. Fluorescence response of different carbon dots to metal ions. The fluorescence spectra change of (**A**) GSH-Asp-CDs. (**B**) GSH-His-CDs. (**C**) GSH-Arg-CDs. (**D**) GSH-Glu-CDs (10 μg/mL, ultrapure water) upon addition of different metal ions (Ag^{+}, Zn^{2+}, Cu^{2+}, Ca^{2+}, Mg^{2+}, Ni^{2+}, Fe^{2+}, Fe^{3+}, Ce^{3+}, Au^{3+}, all at 1 mM) with excitation at 420 nm light.

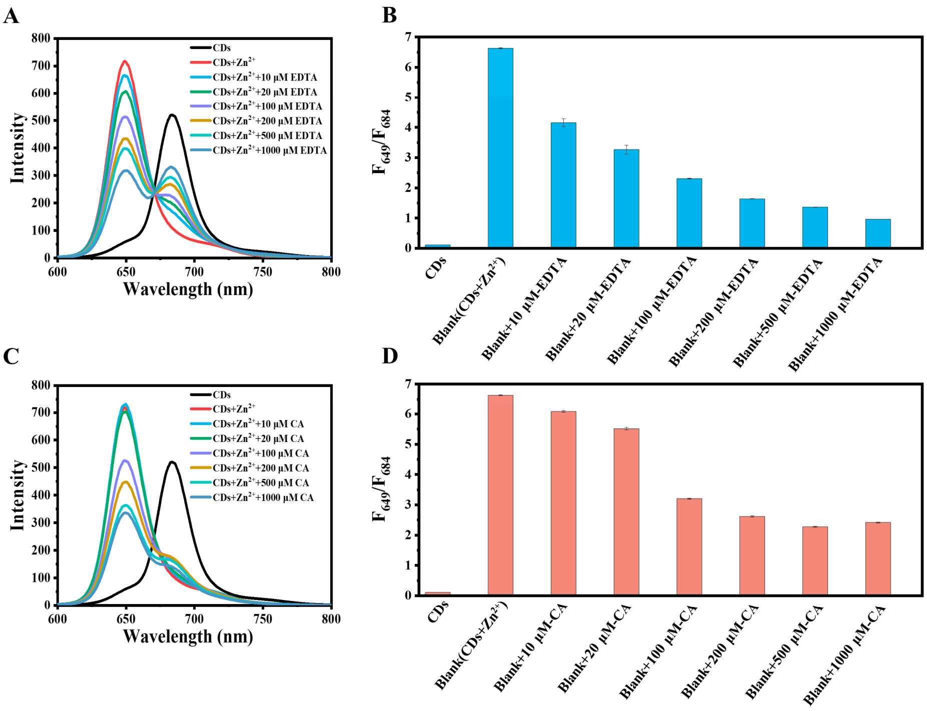

Figure S6. The binding ability between CDs and zinc ions. (**A**) Fluorescence spectra of CDs (10 μg/mL, ultrapure water) under 420 nm excitation after adding different concentrations of EDTA and 20 μM Zinc ions. (**B**) F_{649}/F_{684} of CDs (10μg/mL, ultrapure water) and 20 μM Zinc ions versus EDTA concentration (10-1000 μM). (**C**) Fluorescence spectra of CDs (10 μg/mL, ultrapure water) under 420 nm excitation after adding different concentrations of CA and 20 μM Zinc ions. (**D**) F_{649}/F_{684} of CDs (10 μg/mL, ultrapure water) and 20 μM Zinc ions versus CA concentration (10-1000 μM).

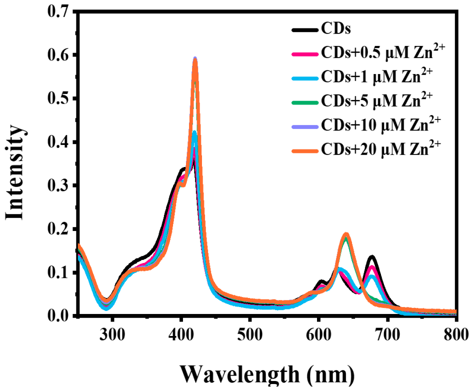

Figure S7. The absorption spectra of CDs. UV-Vis absorption spectra change of CDs (10 μg/mL, ultrapure water) upon addition of different concentrations of zinc ions.

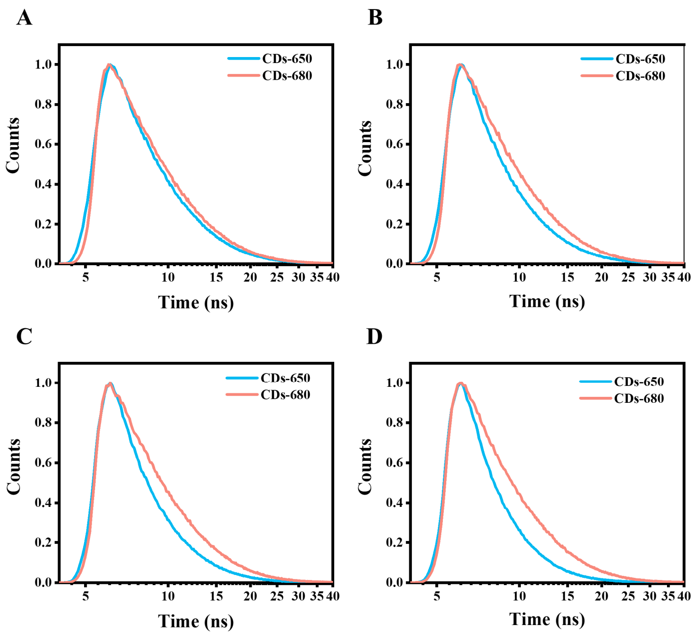

Figure S8. The fluorescence lifetimes of CDs. The fluorescence lifetimes of CDs (10 μg/mL, ultrapure water) at 650 nm and 680 nm for different Zinc ions concentrations. (**A**) 0 μM Zn^{2+}. (**B**) 0.5 μM Zn^{2+}. (**C**) 1 μM Zn^{2+}. (**D**) 2 μM Zn^{2+}.

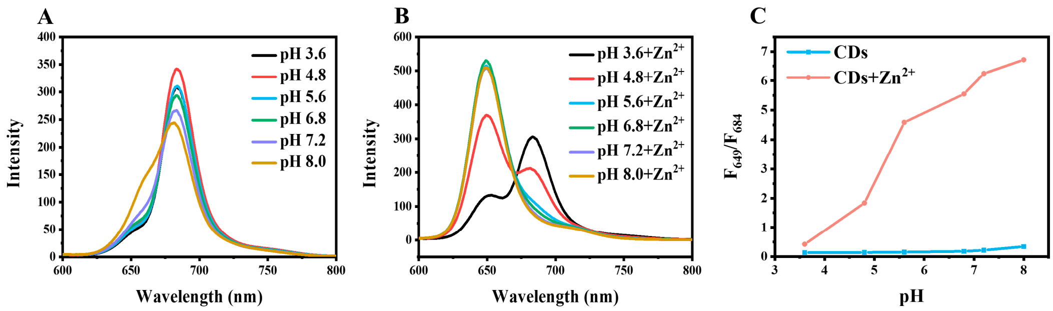

Figure S9. The fluorescence changes of CDs under different pH. (**A**) Fluorescence spectra of CDs (10 μg/mL) under excitation at 420 nm light with different pH values. (**B**) Fluorescence spectra of CDs (10 μg/mL) at different pH values with 20 μM Zinc ions under excitation at 420 nm light. (**C**) F_{649}/F_{684} of CDs (10 μg/mL) with different pH values (blue), F_{649}/F_{684} of CDs (10 μg/mL) at different pH values with 20 μM Zinc ions (red).

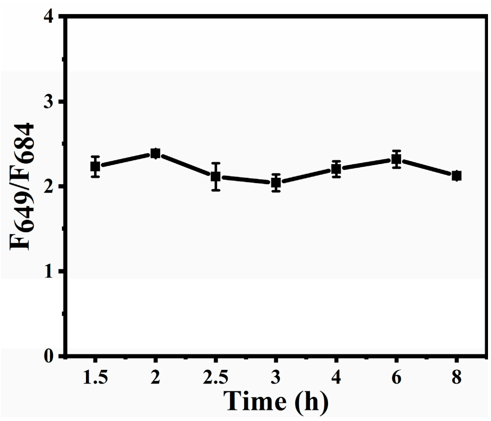

Figure S10. The stability of CDs. The value of the ratio of fluorescence intensity at 649 nm to that at 684 nm (F_{649}/F_{684}) of the CDs (10 μg/mL, ultrapure water) in the presence of zinc ions against time.

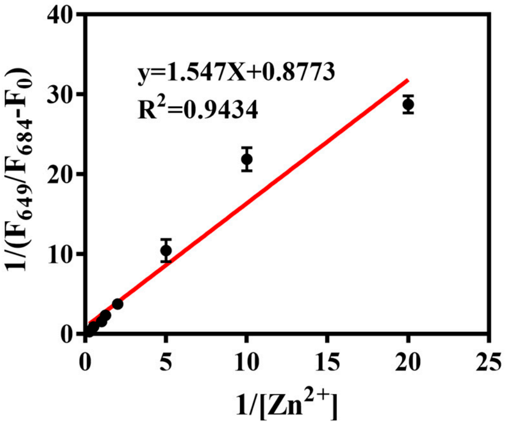

Figure S11. Benesi–Hildebrand plot of the CDs with Zn^{2+}.
